# Translational research in pediatric pulmonary disease, 2017

**DOI:** 10.1186/s40169-017-0142-9

**Published:** 2017-03-01

**Authors:** Bruce K. Rubin

**Affiliations:** Department of Pediatrics, Virginia Commonwealth University School of Medicine, Children’s Hospital of Richmond, 1000 East Broad St., Richmond, VA 23298 USA

## Abstract

As readers of this journal know, translational research is the application of basic science findings to improve clinical care; a process that was once called “bench-to-bedside” research. In my field of pediatric pulmonary disease, the rapid advancement of basic science understanding into improved clinical care has been, to use an appropriate term, breathtaking. In this perspective article, I will describe a few of these advances, as they relate to specific pulmonary diseases.

## Background

As readers of this journal know, translational research is the application of basic science findings to improve clinical care; a process that was once called “bench-to-bedside” research. In my field of pediatric pulmonary disease, the rapid advancement of basic science understanding into improved clinical care has been, to use an appropriate term, breathtaking. In this perspective article, I will describe a few of these advances, as they relate to specific pulmonary diseases.

### Cystic fibrosis (CF)

In many ways, therapeutic development for CF can be considered a paradigm for translational research. Improved understanding of the CF transmembrane ion regulator (CFTR) protein function has led to the development of orally available, small molecule CFTR potentiators and correctors that have dramatically transformed our understanding and care of this disease. The CFTR potentiator, ivacaftor, is the closest that we have come to a “cure” for CF for those patients with class 3, gating CFTR mutations [1]. The combination of ivacaftor and the CFTR corrector, lumacaftor, can decrease the frequency of pulmonary exacerbations in patients with the most common Phe508del, class 2 mutation [2]. There are now many CFTR gene and protein specific therapies being investigated by commercial and academic institutions, in pre-clinical and in more advanced clinical trials. There is also significant interest to see if improving the function of CFTR might affect other airway diseases that are associated with airway inflammation, such as chronic bronchitis.

It is extremely likely that gene editing techniques, such as the CRISPR/Cas9 system, will be first be used to treat diseases like CF or neuromuscular diseases that are associated with single gene abnormalities.

### Asthma

In recent years, we have seen the introduction of biologic therapies for the treatment of severe asthma in older children and adults. These are principally directed at the eosinophilic or TH2 dominant phenotype. There is rich and diverse pipeline of biologics being investigated for improved asthma management [3], and even for the potential prevention of asthma in children with asthma predisposition. Although early intervention with environmental control has not proven to be as effective in preventing asthma, our understanding of early exposure to infections and allergens and how these interact with the child’s genome and gene expression (through epigenetic modification) is giving us a much richer picture of asthma endotypes and to possibly identify children at risk of developing asthma, and how this risk could potentially be modified.

There are intriguing studies underway evaluating alterations of the microbiome, the early immune response, and the interaction between allergens and respiratory viruses to determine if these research applications can successfully be used to treat or prevent the development of this chronic airway disease [4].

### Neuromuscular disease and respiratory compromise

Translational research in patients with neuromuscular disorders has focused on developing drugs that modify the expression of genes associated with diseases such as spinal motor atrophy and muscular dystrophy. Medications like ataluren, a premature termination codon, “read-through” medication, appear to be effective disease modifiers in some of these disorders [5].

### Plastic bronchitis and pulmonary lymphatics

One of the most exciting advances in pediatric pulmonary medicine is recognizing that the pulmonary lymphatics play an important role in the development of pulmonary disease. Plastic bronchitis is a syndrome where the airways fill with firm, cohesive material that can completely obstruct. It is most common in children with congenital heart disease and single ventricle physiology, although only a small minority of children with single ventricle physiology will develop plastic bronchitis [6]. Innovative work by Dori and Itkin, out of Philadelphia, has demonstrated that the children who develop plastic bronchitis with congenital heart disease, have aberrant lymphatic drainage to the thoracic duct, and that this aberrant drainage can be corrected by lymphatic intervention, gluing shut the abnormal vessels [7]. In almost all patients that are so treated, this has been life changing. Interest in the role of pulmonary lymphatics has also led to novel discoveries as to how this “third” circulatory system, in the lungs, can affect diseases such as chronic lung disease of the newborn.

### Childhood interstitial lung diseases (chILD)

The interstitial lung diseases of childhood, so called chILD, until fairly recently were classified based upon histologic appearance and clinical outcomes and although these metrics are still useful; identification of the genetic causes of many of these diseases is making classification more precise and helping to identify potential therapeutic targets. Moreover, in the case of surfactant metabolism disorders of the neonate and infant, what were once considered histologically distinctly disorders, e.g. pulmonary alveolar proteinosis (PAP), nonspecific interstitial pneumonitis, diffuse interstitial pneumonitis, and chronic pneumonitis of infancy are now recognized to have related underlying genetic mechanisms making it possible to identify some patients with these disorders by genetic testing rather than by lung biopsy [8].

Using PAP as an example, it is now recognized that the severe, neonatal form is caused by deficiency in the hydrophobic surfactant apoprotein (Sp)B. Abnormalities in the adenosine binding cassette subtype A3 (a phospholipid transporter) can also cause PAP and abnormalities in SpC can cause later onset ILD in the child and in adulthood. The more common, but still quite rare adult onset form of PAP is usually caused by autoantibodies to the granulocyte–macrophage colony stimulating factor (GM-CSF) essential for maturation of alveolar macrophages; the cell responsible for surfactant recycling in the acinus. Understanding the key role of GM-CSF in surfactant metabolism permitted the identification of a juvenile onset PAP caused by abnormalities in the GM-CSF receptor [9]. This, in turn, has allowed the development of novel therapies for these serious diseases.

This is a brief overview of how basic research is being rapidly applied to dramatically transform the clinical care of children with pulmonary disease. With tools that allow us to better understand the role of the genome, bio-informatics for managing massive databases, better phenotyping and genotyping of diseases, and an understanding of how social and environment influences can alter the epigenetic expression of genes and proteins we are likely to see dramatic advances in clinical care in the next decade.

## Abbreviations

CF: cystic fibrosis
CFTR: CF transmembrane ion regulator
chILD: interstitial lung diseases of childhood
GM-CSF: granulocyte macrophage colony stimulating factor
PAP: pulmonary alveolar proteinosis
TH2: T-helper cell type 2
